# A giant invasive macroprolactinoma with recurrent nasal bleeding as the first clinical presentation: case report and review of literature

**DOI:** 10.1186/s12902-023-01345-y

**Published:** 2023-05-12

**Authors:** Danting Li, Yan Wang, Huiwen Tan, Peiqiong Luo, Yerong Yu

**Affiliations:** 1 Division of Endocrinology and Metabolism, West China Hospital of Sichuan University, Chengdu, 610041 P.R. China; 2 Department of Health Management, Health Management Center, General Practice Center, West China Hospital of Sichuan University, Chengdu, 610041 P.R. China

**Keywords:** Giant macroprolactinoma, Nasal bleeding, Pituitary adenoma, Invasive, Bromocriptine

## Abstract

**Background:**

Giant prolactinoma (> 4 cm in dimension) is a rare disorder. Invasive macroprolactinoma has the potential to cause base of skull erosion and extend into the nasal cavity or even the sphenoid sinus. Nasal bleeding caused by intranasal tumor extension is a rare complication associated with invasive giant prolactinoma.

We report a case of giant invasive macroprolactinoma with repeated nasal bleeding as the initial symptom.

**Case presentation:**

A 24-year-old man with an invasive giant prolactinoma in the nasal cavity and sellar region who presented with nasal bleeding as the initial symptom, misdiagnosed as olfactory neuroblastoma. However, markedly elevated serum prolactin levels (4700 ng/mL), and a 7.8-cm invasive sellar mass confirmed the diagnosis of invasive giant prolactinoma. He was treated with oral bromocriptine. Serum prolactin was reduced to near normal after 6 months of treatment. Follow-up magnetic resonance imaging showed that the sellar lesion had disappeared completely and the skull base lesions were reduced.

**Conclusion:**

This case is notable in demonstrating the aggressive nature of untreated invasive giant prolactinomas which can cause a diagnostic difficulty with potential serious consequences. Early detection of hormonal levels can avoid unnecessary nasal biopsy. Early identification of pituitary adenoma with nasal bleeding as the first symptom is particularly important.

**Supplementary Information:**

The online version contains supplementary material available at 10.1186/s12902-023-01345-y.

## Background

Invasive giant prolactinoma is one of the rare subtype of prolactin (PRL)-secreting pituitary neuroendocrine tumors (Pit-NETs), measuring > 40 mm, accounting for only 0.5% of all Pit-NETs, and 4.4% of all prolactinomas, often presenting with headaches, vision loss, hyperprolactinemia, hypopituitarism as the first clinical manifestations [1–3]. Therapeutic goals for prolactinoma include normalization of prolactin levels, significant tumor reduction, and restoration of function of the anterior and posterior pituitary gland. Cabergoline is the first-line treatment. For untreated giant prolactinomas nasal bleeding as initial symptom is extremely rare and often misdiagnosed. Here, we report the case of a 24-year-old young man with a giant prolactinoma in the nasal cavity and sellar area who presented with nasal bleeding as the first symptom.

## Case presentation

### Case report

A 24-year-old young man presented initially with nasal bleeding as the first symptom. He was admitted to West China Hospital of Sichuan University due to “intermittent nasal bleeding for 2 years and vision loss for 6 months”.

The patient had a 2-year history of infrequent nasal bleeding and intermittent episodes of nasal bleeding. He had reported no nasal obstruction or dizziness, and nasopharyngoscopy in other hospitals had revealed no abnormalities. He had no nausea, anorexia, weakness, weight gain, gynecomastia, fatigue, or other symptoms, and he was taking no medication. However, 6 months before presentation at our hospital, following repeated epistaxis the patient developed blurred vision in both eyes, and his visual field decreased and gradually worsened. He reported intermittent mild headaches but no sinus pain, numbness, tingling, or constitutional symptoms. The patient did not report sexual dysfunction, including decreased libido or erectile dysfunction. His family history was unremarkable and he had no history of traumatic injury or brain surgery before admission.

On physical examination, his temperature was 36.5 °C, his pulse was 83 beats per minute, his blood pressure was 119/75 mmHg, and his respiratory rate was 19 breaths per minute. His height was 169.0 cm, his weight was 67.7 kg, and body-mass index (BMI, the weight in kilograms divided by the square of the height in meters) was 23.7 kg/m^2^. He did not have acromegalic or cushingoid features. The remainder of the examination was normal.

**Fig. 1:**
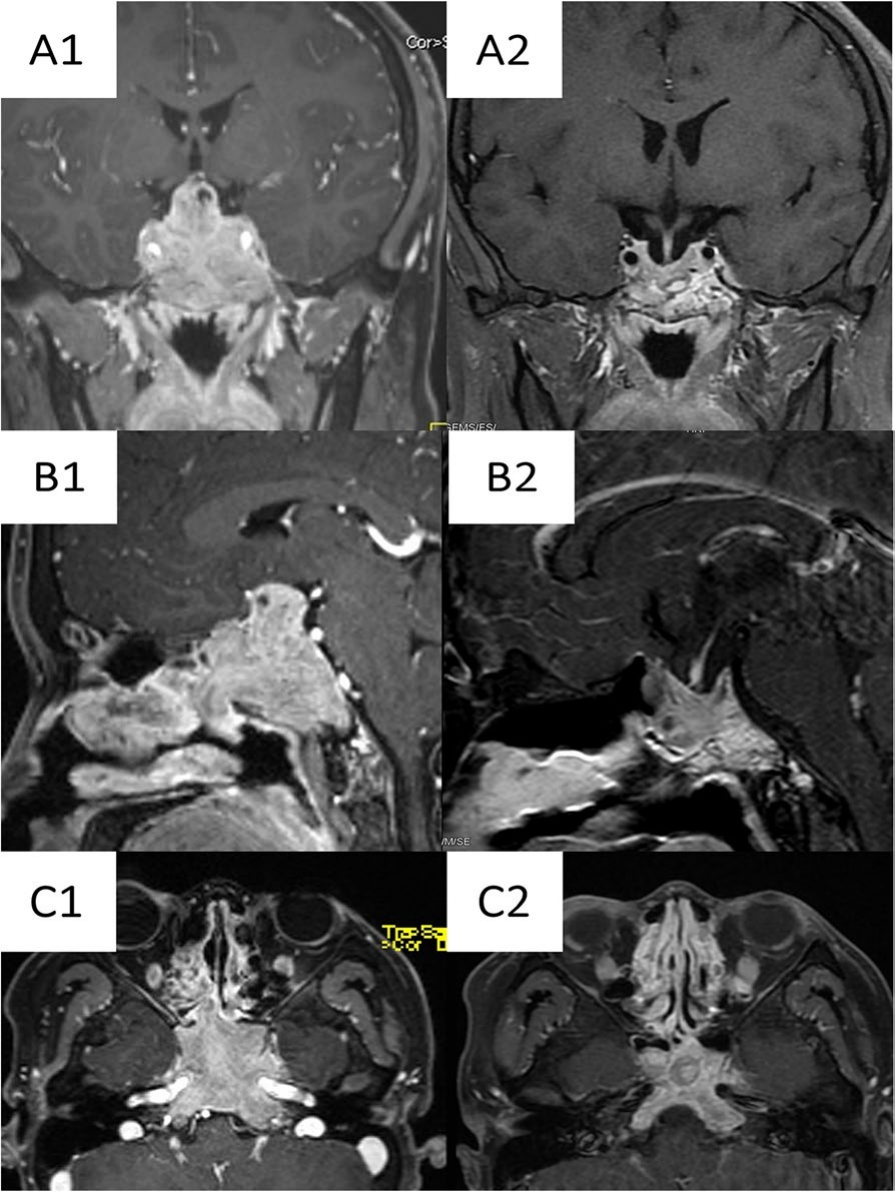
Pituitary enhanced magnetic resonance imaging (MRI) showing tumor shrinkage after treatment with a dopamine agonist in the young patient with a macroprolactinoma. MRI scans obtained before dopamine agonist therapy, showing a large pituitary mass in coronary, sagittal and axial station (A1, B1 and C1, respectively) and after 1 year of treatment, with marked reduction of the mass (A2, B2 and C2, respectively). Before treatment with bromocriptine, an irregular mass about 7.8 cm × 4.6 cm × 5.0 cm was observed on the saddle. It invades the sphenoid and cavernous sinuses, with encasement of the carotid arteries and optic chiasm compression

Nasopharyngeal magnetic resonance imaging (MRI) and nasopharyngeal biopsy were performed. Pituitary MRI showed an irregular, abnormal signal mass in the nasal cavity, sphenoid sinus, and sellar region, about 7.8 × 4.6 × 5.0 cm in size (Fig. 1A, B, C). The anterior pituitary gland exhibited high signal on T1-weighted images and/or low signal on T2-weighted images. The mass enhanced heterogeneously after gadolinium. It invades the sphenoid and cavernous sinuses, with encasement of the carotid arteries and optic chiasm compression. Electronic rhinopharyngoscopy showed chronic congestion of the nasal mucosa on both sides and new organisms in the right olfactory cavity. There was purulent attachment in the nasopharyngeal region, chronic congestion, and swelling of the nasopharyngeal mucosa. A biopsy of the nasal cavity suggested olfactory neuroblastoma of the right olfactory cleft. Immunohistochemistry analysis indicated Syn ( +), CK ( +), Ki67 30%, CgA (-), EMA (-), S100(-), HMB (-), and LCA (-).

### Diagnostic assessment

**Fig. 2:**
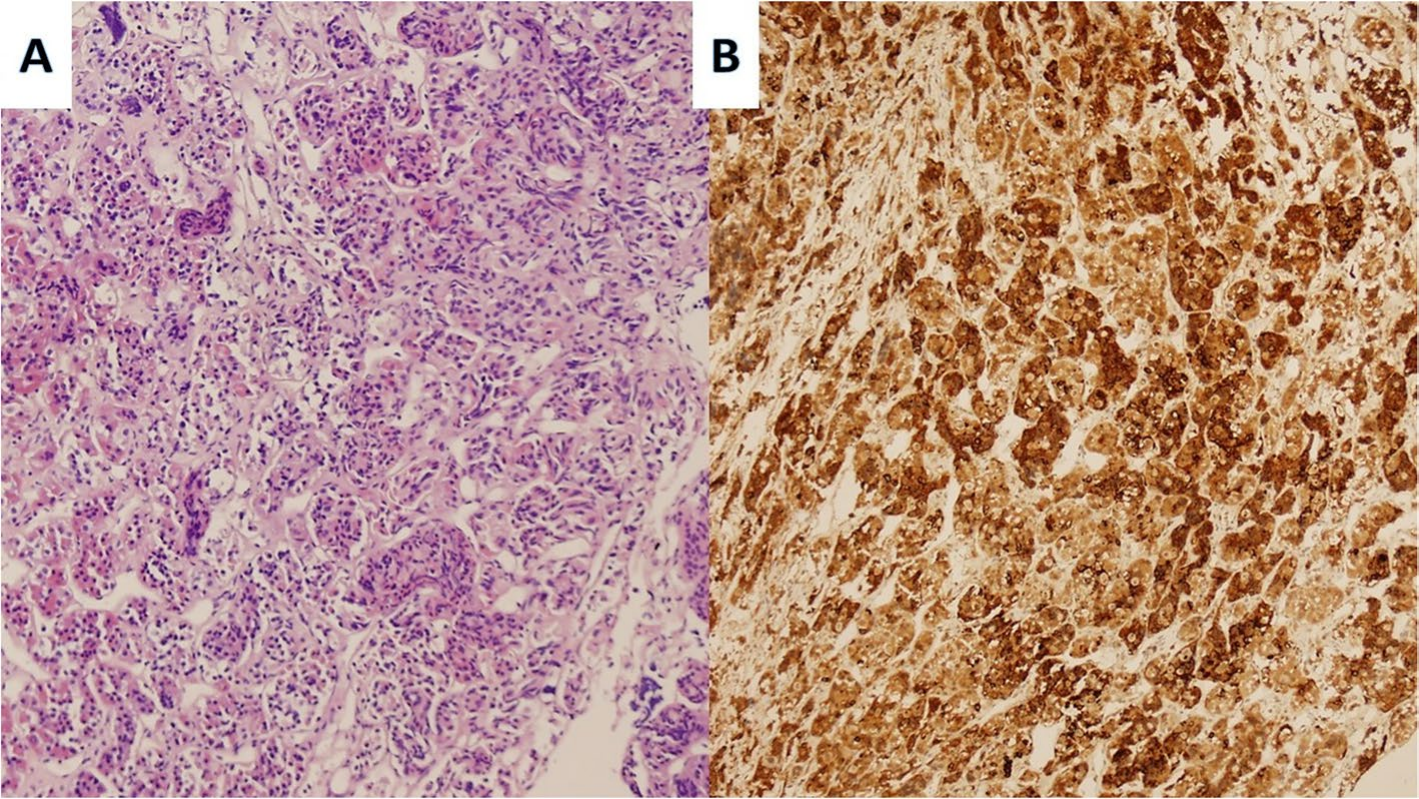
The biopsy image of a patient with invasive giant prolactinoma after hematoxylin–eosin stain (**A**: magnification × 400). Immunostaining showed positive staining for PRL (**B**: magnification × 400)

The patient was diagnosed with olfactory neuroblastoma and was scheduled to undergo surgery, supplemented by radiotherapy and chemotherapy. The main reason for the impaired vision and visual field was considered to be that the suprasellar lesions were compressing the optic chiasm. To save vision and the visual field, otolaryngologists planned to work with neurosurgeons to remove the upper lesions of the saddle in order to decompress the optic nerve, followed by radiotherapy of the nasopharynx. However, after carefully reading the MRI results, we determined that the lesion was invasive and mainly located in the sphenoid sinus and the cavernous sinus and saddle. There was no obvious space in the ethmoid sinus or nasal cavity, which is not completely consistent with the characteristics of olfactory neuroblastoma. Routine preoperative examination of pituitary and target-glands related hormones showed the initial PRL level of this patient was greater than 200 ng/mL, and the measured value after dilution was as high as 4700 ng/mL, with normal thyroid-stimulating hormone (TSH), free thyroxine (FT4), luteinizing hormone (LH), follicle-stimulating hormone (FSH), testosterone (T) and cortisol levels (Table 1). Combined with the patient’s medical history, physical symptoms, imaging and laboratory tests, the diagnosis of giant prolactinoma was considered. We then reviewed the pathological section and added pituitary tumor immunohistochemical staining (Fig. 2A, B).

**Table 1:** Laboratory data of the patient with invasive giant prolactinoma

Items	Results	Reference value and normal range	Units
GH	0.40	0.03–2.47	ng/ml
IGF-1	145.35	111.4–256.8	ng/ml
TSH	1.55	0.27–4.2	mIU/L
FT3	4.01	3.60–7.50	pmol/L
FT4	13.56	12.0–22.0	pmol/L
ACTH	24.30	5.0–78.0	ng/L
PTC	496.90	147.3–609.3	nmol/L
LH	2.6	1.7–8.6	mIU/mL
FSH	3.9	1.5–12.4	mIU/mL
E2	9.59	25.8–60.7	pg/mL
P	0.23	0.05–0.149	ng/mL
PRL	> 4700	4.6–21.4	ng/mL
T	2.77	2.49–8.36	ng/mL
DHEAs	1.59	1.2–8.98	umol/L

*PRL* Preoperative routine examination of pituitary and target-glands related hormones, serum prolactin, *GH* Growth hormone, *IGF-1* Insulin-like growth factor-1, *TSH* Thyroid-stimulating hormone, *FT3* Free triiodothyronine, *FT4* Free thyroxine, *ACTH* Adrenocorticotropic hormone, *PTC-8:00* 8:00 am plasma cortisol, *LH* Luteinizing hormone, *FSH* Follicle-stimulating hormone, *E2* Estradiol, *P* Progesterone, *T* Testosterone, *DHEA-S* Dehydroepiandrosterone sulfate

He was administered oral bromocriptine at an initial dose of 2.5 mg, once a day because of difficulties in obtaining cabergoline in Chengdu. After 3 days, the dose was increased to 2.5 mg twice a day. No nausea, vomiting, or other discomfort followed. The oral bromocriptine was gradually increased after 1 week to 2.5 mg, 3 times per day. He has tolerated the bromocriptine well without any adverse events. The PRL levels were reviewed each month, and the MRI (plain scan + enhancement) was repeated at 6 months and 12 months after treatment. Prolactin levels had reached normal or near-normal level within approximately 6 months. After 12 months of follow-up, the patient’s visual acuity and visual field improved significantly, and PRL had gradually normalized. The nasopharynx was examined, and we found that the nasopharynx mucosa was chronically congested; the nasopharynx, the double pharyngeal crypt, the eustachian tube bulge, and the surface of the pharynx were smooth. Magnetic resonance imaging of the sellar region showed a significant reduction in the size of the giant macroprolactinoma.

## Discussion and conclusion

We present a case of a 24-year-old male patient diagnosed with a giant prolactinoma invading the nasal cavity. Rarely for an invasive giant prolactinoma, the initial presenting symptom was nasal bleeding. Prolactinoma is one of the most common pituitary adenomas among Pit-NETs, with a prevalence of 6–10 to 60 cases per 100,000 patients. Prolactinomas are categorized based on their size, with microadenomas measuring < 1 cm, macroadenomas measuring > 1 cm, and giant prolactinomas, which are characterized by size > 4 cm, and PRL levels > 1,000 ng/mL [1]. Giant prolactinomas are rare tumors accounting for 0.5–1% of all pituitary tumors and 2–3% of prolactinomas [4].

Most giant prolactinomas are invasive, and can have suprasellar extension, invasion to cavernous and sphenoid sinuses and clivus and rarely posteriorly into the brainstem, but they do not have biological characteristics of malignant tumors. Patients with giant pituitary prolactinoma may have a higher incidence of visual disturbances [4]. Other symptoms of presentation may be hypogonadism, and headaches [4, 5]. Prolactinomas in men are often invasive tumors that grow rapidly, and they appear to have a more aggressive nature compared to females. The PRL level is high before treatment, and the size of the adenoma is directly proportional to the plasma PRL concentration [1]. The clinical manifestations of male hyperprolactinemia include erectile dysfunction, decreased libido, and infertility.

The patient had normal libido, no sarcopenia and testosterone levels were normal. The possible explanation is that this patient retains an intact pituitary–gonadal hormonal axis. There have been many reports of this phenomenon, such as Shimon et al., which reported that 40% of men presenting with prolactinomas presented with normal or borderline testosterone levels [6]. Ono M et al. reported a series of 28 men with prolactinomas, about 42% of these patients had normal pretreatment testosterone [7]. While Sibal et al. reported that 23% male patients with macroprolactinomas had unaffected gonadotropin-testosterone axis [8].

Pituitary adenomas presenting with nasal bleeding as the first symptom are extremely rare. In 1989, Van der Mey reported three patients with pituitary adenoma invading the nasal cavity. Of those patients, two with pituitary adenoma first presented with epistaxis [9]. Similar case reports have been published in the past decade (Table 2) [9–17]. Because of the atypical symptoms, such patients are easily misdiagnosed with otolaryngologic conditions during initial hospital visits. Chaurasia et al. reported a case of a large invasive prolactinoma in a child with nasal bleeding as the first manifestation [11], in which otolaryngologic treatment was ineffective; 3 months later, due to decreased vision in the right eye, MRI revealed a large footprint in the saddle area. Only then was the pituitary adenoma diagnosed.

**Table 2:** Clinical profiles of the patient with invasive pituitary adenomas presenting with nasal bleeding

Authors	Age/gender	Visual loss	Other symptoms	Department	PRL	CT	MRI	Histopathologic evaluation with immunohistochemistry (IHC)	Outcome
Van Der Mey AG et al., 1989 [9]	54/M	N (A slight sixth nerve paresis of the left eye)	Frontal headache, nasal obstruction, rhinorrhea, impotent	The otolaryngology department	582 to 820 μg/L (reference, < 12 μg/L)	A mass extending into the ethmoid and sphenoid sinuses, nasal cavity, and naso­pharynx	The same tumor extension as CT	Prolactinoma	In good condition
Van Der Mey AG et al., 1989 [9]	34/M	Y (Hemianopsia and a slightly decreased visual acuity)	Nasal obstruction	NA	7.5 μg/L	A sellar mass invading the third ventricle and nasal cavity	NA	Polyhormonal tumor(PRL and GH)	In good condition
Van Der Mey AG et al., 1989 [9]	47/F	Y (Decreased visual acuity and diplopia	Nasal obstruction, occipital and frontal headache	NA	20 μg/L	A mass invading nasal cavity and maxillary sinus	NA	A chromophobe adenoma, and immunochemistry showed no hormone-containing granules	In good condition
Godey B et al., 1999 [14]	45/M	N	Nasal obstruction	NA	Elevated	A mass invading the cavernous sinus	A sellar mass	Polyhormonal tumor(PRL, FSH and LH)	The MRI confirmed the regression of the tumor
Godey B et al., 1999 [14]	39/M	Y	Intermittent pain over the maxillary and frontal sinuses, a fever and purulent rhinorrhea	NA	Elevated	NA	A sellar mass	Prolactinoma	The tumor had decreased in size by 50%
Hofman R et al., 2010 [10]	74/F	N	NA	The otolaryngology department	697,000 mU/l (reference, < 500 mU/l)	NA	A mass (> 6 cm) invading the sphenoid sinus and nasopharynx	Prolactinoma	In good condition
Chaurasia PK et al., 2011 [11]	13/M	Y (No vision in right eye)	Nasal obstruction	NA	203.7(reference, 86–324 mic IU/ml.)	NA	A sellar-suprasellar mass eroding bilateral optic canal, nasopharynx, nasal cavity, sphenoid sinuses, et al	Prolactinoma	In good condition
Sahoo JP et al., 2015 [12]	35/F	N	Headache and nasal obstruction	The otolaryngology department	7443 μg/L (reference, < 20 μg/L)	NA	A sellar and infra-sellar mass extending into the nasopharynx	Prolactinoma	In good condition
Imamura J et al., 1998 [13]	72/F	Y (Only light was distinguishable in right eye)	Headache and nausea	The otolaryngology department	6800 ng/mL (reference, 1.4–14.6 ng/mL)	A giant mass in the sella turcica extending into the cavernous sinus and suprasellar cistern, enclosing a large intracavernous aneurysm of the internal carotid artery	NA	Prolactinoma	Died of massive bleeding from aneurysmal rupture
Kleinschmidt-Demasters BK et al., 1998 [15]	67/M	Y (Sudden onset visual loss in right eye)	Ophthalmoplegia	NA	NA	NA	Sellar /suprasellar mass, peripheral rim enhancement with gadolinium	Null cell adenoma	NA
Ghannam NN et al., 1999 [16]	41/M	Y (Bilateral temporal visual defects)	Right nasal obstruction, classical symptoms of hypothyroidism, headache, right earache	NA	< 2 μg/L (reference, 2.1–17.7 μg/L)	A mass involving the nasal cavity, sphenoid sinus, and the sella	NA	TSH-secreting pituitary tumor	NA
Das CJ et al., 2006 [17]	43/M	N	NA	The otolaryngology department	NA	NA	Sphenoid and ethmoid sinuses mass extending into the nasopharynx, bilateral cavernous sinuses, and right petrous apex; empty sella	Nonfunctioning invasive pituitary adenoma	NA

*Abbreviations*: *F* Female, *M* male, *NA* Not available, *PRL* Prolactin, *GH* Growth hormone, *FSH* Follicle-stimulating hormone, *LH* Luteinizing hormone, *SGLA* Sparsely granulated lactotroph adenoma, *ASCA* acidophil stem cell adenoma

In the present case, our patient had repeated nasal discharge as the first symptom, and nasal laryngoscopy revealed a new organism in the right olfactory sulcus. Olfactory neuroblastoma was suspected. However, the significantly increased serum PRL(> 200 ng/ml) excluded the possibility. The initial PRL level of this patient was greater than 200 ng/mL, and the measured value after dilution was as high as 4700 ng/mL. The main reason for this phenomenon may be the hook effect, which should be considered in all cases of large prolactinomas with normal or mildly elevated PRL levels [18]. The hook effect mainly appeared in the monoclonal sandwich assays, which could be avoided by repeated measurements of PRL after a 1:100 serum sample dilution [19].The serum PRL level is often related to the tumor size. Most patients with serum PRL levels > 250 ng/mL have macroprolactinomas (diameter > 10 mm). Of course, there are exceptions where the level of PRL is inconsistent with the size of the pituitary tumor. It is worth noting that a sellar mass and prolactin levels more than 200–250 ng/ml are important criterion for the diagnosis of prolactinoma. The use of bromocriptine to treat the lesions rapidly reduced their size; it was found that serum PRL decreased to the normal level, and there was also no recurrence of the lesions and no obvious abnormalities on the nasopharyngeal examination, which supported the clinical diagnosis.

When a pituitary adenoma invades the nasal cavity, it needs to be differentiated from some primary nasal tumors, such as nasopharyngeal malignant lymphoma and olfactory neuroblastoma [20], although typical cases have certain characteristics and there are other local clinical manifestations. Imaging studies and pituitary hormone levels are very important to identify pituitary adenomas. Histopathological findings could be misleading. If tumor cells are poorly differentiated and have more mitotic figures, a pituitary adenoma may be misdiagnosed as neuroblastoma, undifferentiated carcinoma, or malignant lymphoma [21]. Hyrcza et al. reviewed a series of nonectopic pituitary adenomas presenting as sinonasal or nasopharyngeal masses [22]. Of the 13 cases reviewed, the initial diagnosis by biopsy of the nasopharyngeal tumor was incorrect in 3 cases, with 2 tumors misdiagnosed as olfactory neuroblastoma and 1 as neuroendocrine carcinoma. They were also treated with surgery or chemoradiotherapy, 2 of them had a poor response to treatment. Pathology review of the original biopsy by pituitary-specific immunohistochemical stains confirmed the diagnosis of gonadotroph adenoma, sparsely granulated lactotroph adenoma, pituitary acidophil stem cell adenoma, respectively. Van der Lely et al. [23] also reported six cases of nasal tumors in 1992; after repeated pathological examination, four cases were diagnosed as malignant undifferentiated carcinomas, and the other two were diagnosed as neuroblastoma. By immunohistochemistry, the corresponding antibodies were used to detect PRL, FSH, LH and GH, combined with serum pituitary hormone levels; four cases of pituitary adenoma were confirmed. In the present case, a biopsy of the lesion in the nasal cavity was performed, suggesting an olfactory neuroblastoma, which was diagnosed as an invasive prolactinoma by high prolactin levels as well as immunohistochemistry that stained for prolactin, similar to a previous case in the literature [9]. Therefore, when the pathology and clinical diagnosis are not consistent, comprehensive analysis should be combined with clinical data. It is important to note that well-differentiated neuroendocrine tumors of the nasopharyngeal mucosa overlap with pituitary adenomas in morphology and immunohistochemistry. The distinction requires pituitary-specific immunohistochemical stains, such as the transcription factors Pit-1, Tpit, and SF-1, as well as pituitary hormones [22].

The goal of treatment for large invasive prolactinomas is to reduce tumor volume and serum PRL levels. In the case presented here, the MRI of the saddle area showed a large tumor; the pituitary PRL level or pituitary imaging had not been previously screened. The level of PRL found in this case was significantly higher than normal. At present, the first-line treatment for giant prolactinomas is medical with dopamine agonists [24]. A meta-analysis from Zamanipoor Najafabadi AH [25] show that surgery is not the preferred treatment for invasive prolactinoma because postoperative remission rates are less favorable. Dopamine receptor agonists are effective at normalizing PRL levels and tumor shrinkage [26–28]. The mechanism of action is selective agonism of dopamine 2 receptor on PRL cell membranes and inhibition of PRL mRNA gene expression and PRL cell metabolism, resulting in reduced PRL synthesis secretion and tumor volume shrinkage [29]. Clinically, bromocriptine and cabergoline are commonly used. It’s worth noting that rapid dose escalation may lead to massive tumor shrinkage with a potential risk of apoplexy or cerebrospinal fluid leak [30–32].

In the case reported here, since it was difficult for our patient to obtain cabergoline at that time, oral bromocriptine was preferred. The PRL was significantly reduced after 6 months by gradually increasing the dose. A follow-up MRI scan showed that the lesions in the sellar region had nearly totally vanished, and the lesions in the skull base and nasal cavity were reduced. Additionally, the patient’s visual acuity improved, and there was no more nasal hemorrhage.

In summary, pituitary adenomas rarely invade the nasopharyngeal cavity, especially with repeated nasal bleeding as a first reported symptom. These pituitary adenomas presenting as nasopharyngeal masses can cause a diagnostic difficulty with potential serious consequences, such as adopt unnecessary treatments and delay optimal treatments, increase the potential side effects and economic burden. Therefore, we emphasize the importance of radiologic studies and hormone detection. Once the diagnosis of prolactinoma is established, dopamine agonist should be the first choice for treatment.

## Supplementary Information

**Additional file 1.**

## Data Availability

The original contributions presented in the study are included in the article/supplementary material. Further inquiries can be directed to the corresponding author.

## Abbreviations

PRL: Prolactin
Pit-NETs: Pituitary neuroendocrine tumors
BMI: Body-mass index
MRI: Magnetic resonance imaging
TSH: Thyroid-stimulating hormone
FT4: Free thyroxine
FT3: Free triiodothyronine
LH: Luteinizing hormone
FSH: Follicle-stimulating hormone
T: Testosterone
GnRH: Gonadotropin-releasing hormone
GH: Growth hormone
IGF-1: Insulin-like growth factor-1
ACTH: Adrenocorticotropic hormone
PTC: Plasma cortisol
E2: Estradiol
P: Progesterone
DHEA-S: Dehydroepiandrosterone sulfate
SGLA: Sparsely granulated lactotroph adenoma
ASCA: Acidophil stem cell adenoma
