# Supplementary figures and images for: A giant invasive macroprolactinoma with recurrent nasal bleeding as the first clinical presentation: case report and review of literature
Source: BMC Endocr Disord. 2023 May 12;23:107. doi: 10.1186/s12902-023-01345-y (PMC10176701; doi:10.1186/s12902-023-01345-y)

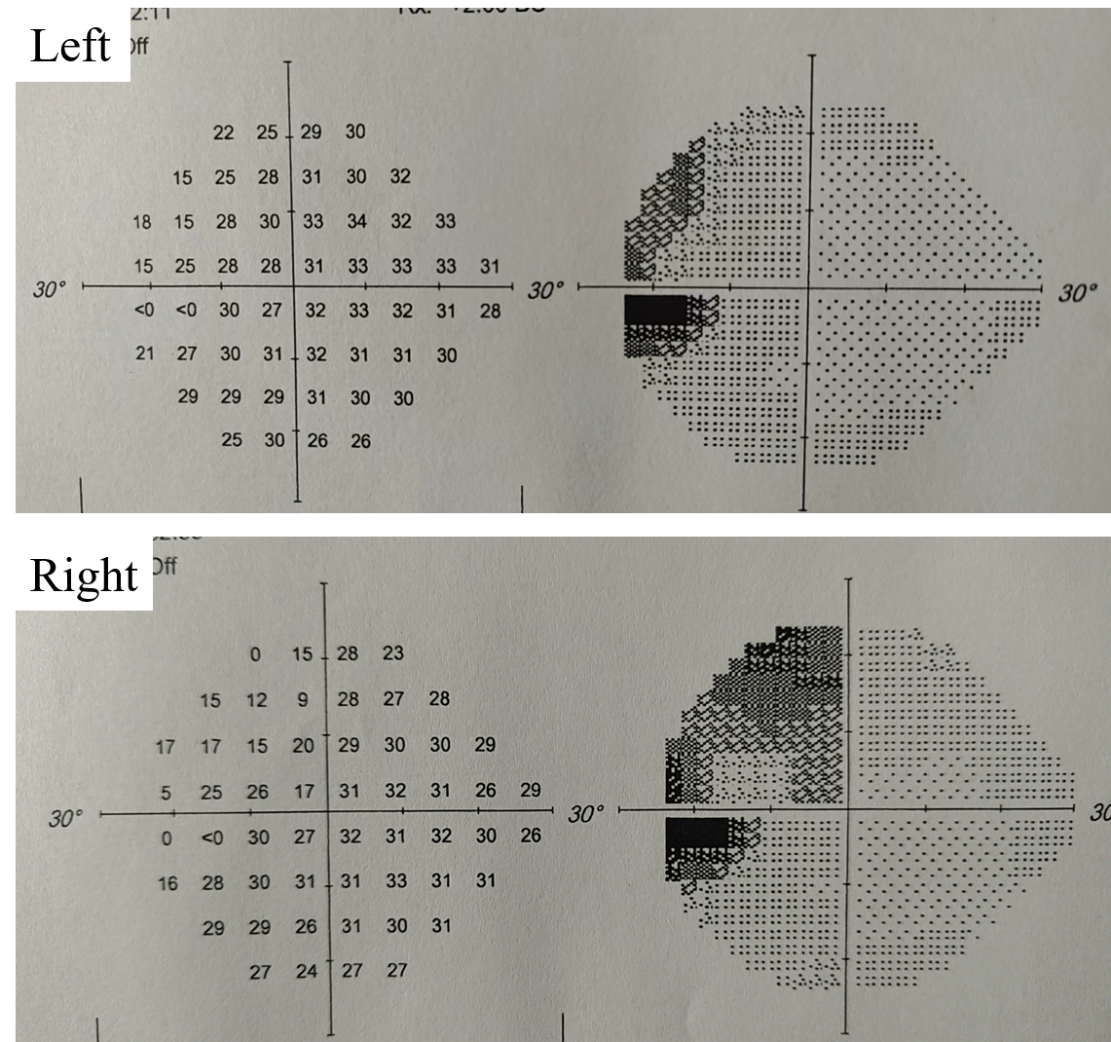

Supplement: Supplementary file 1 — Additional file 1. [file 12902_2023_1345_MOESM1_ESM.zip › bmc vision1R3.tif]

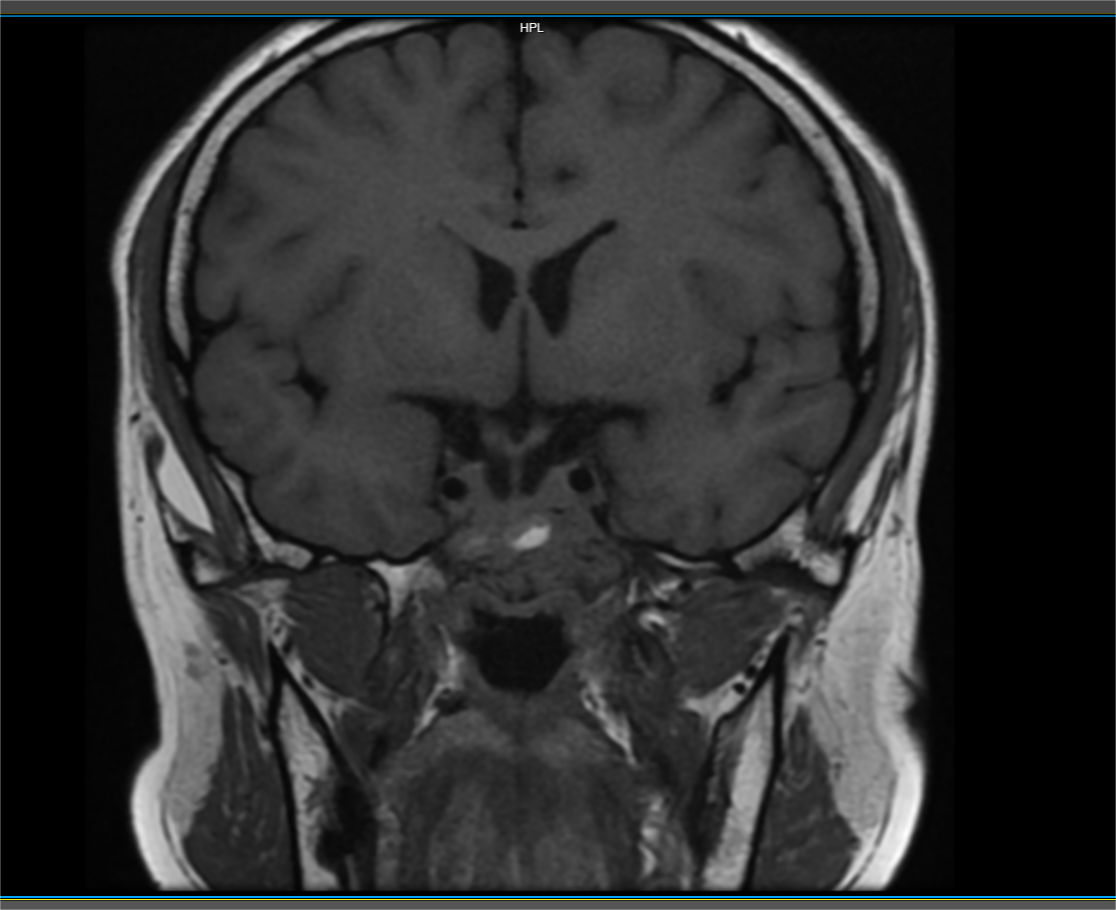

Supplement: Supplementary file 1 — Additional file 1. [file 12902_2023_1345_MOESM1_ESM.zip › fig 2(t2)R3.png]

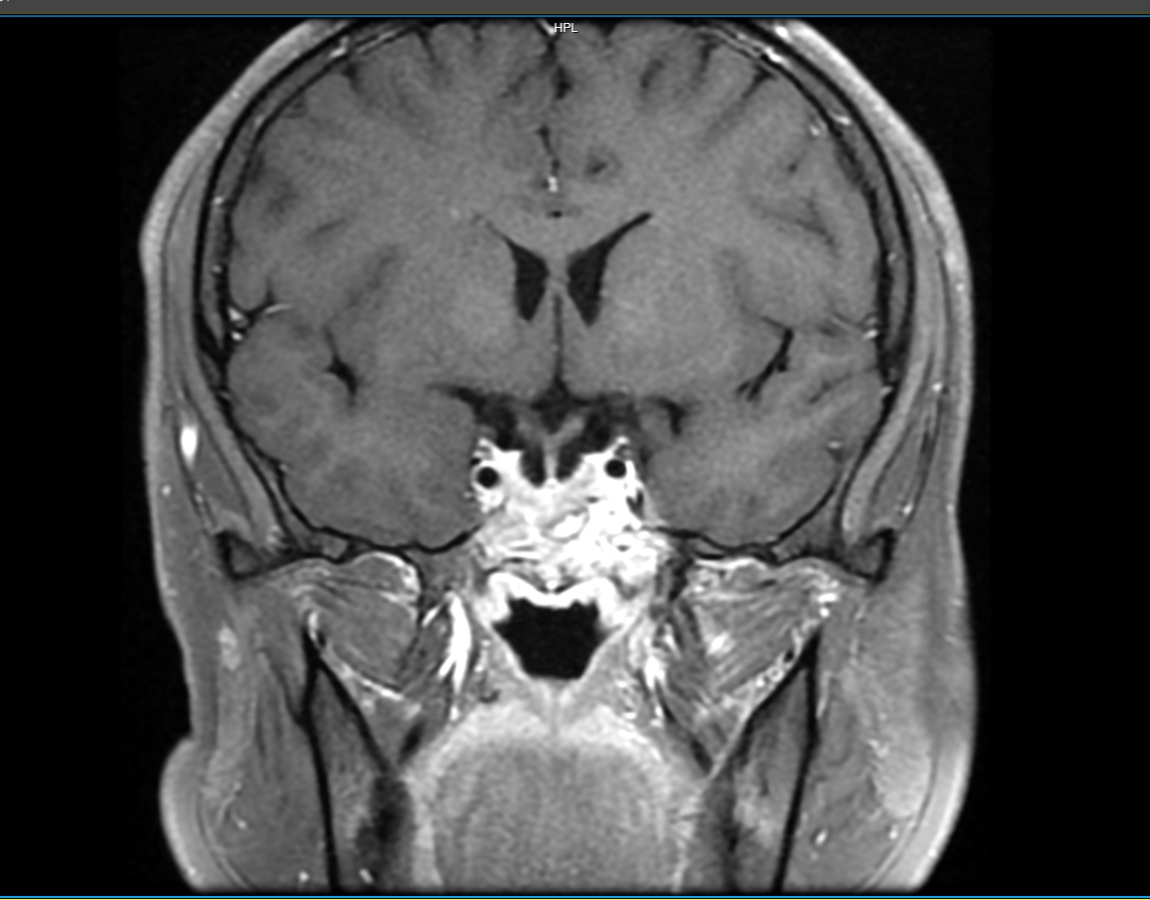

Supplement: Supplementary file 1 — Additional file 1. [file 12902_2023_1345_MOESM1_ESM.zip › fig(1)fuyingyingR3.jpg]

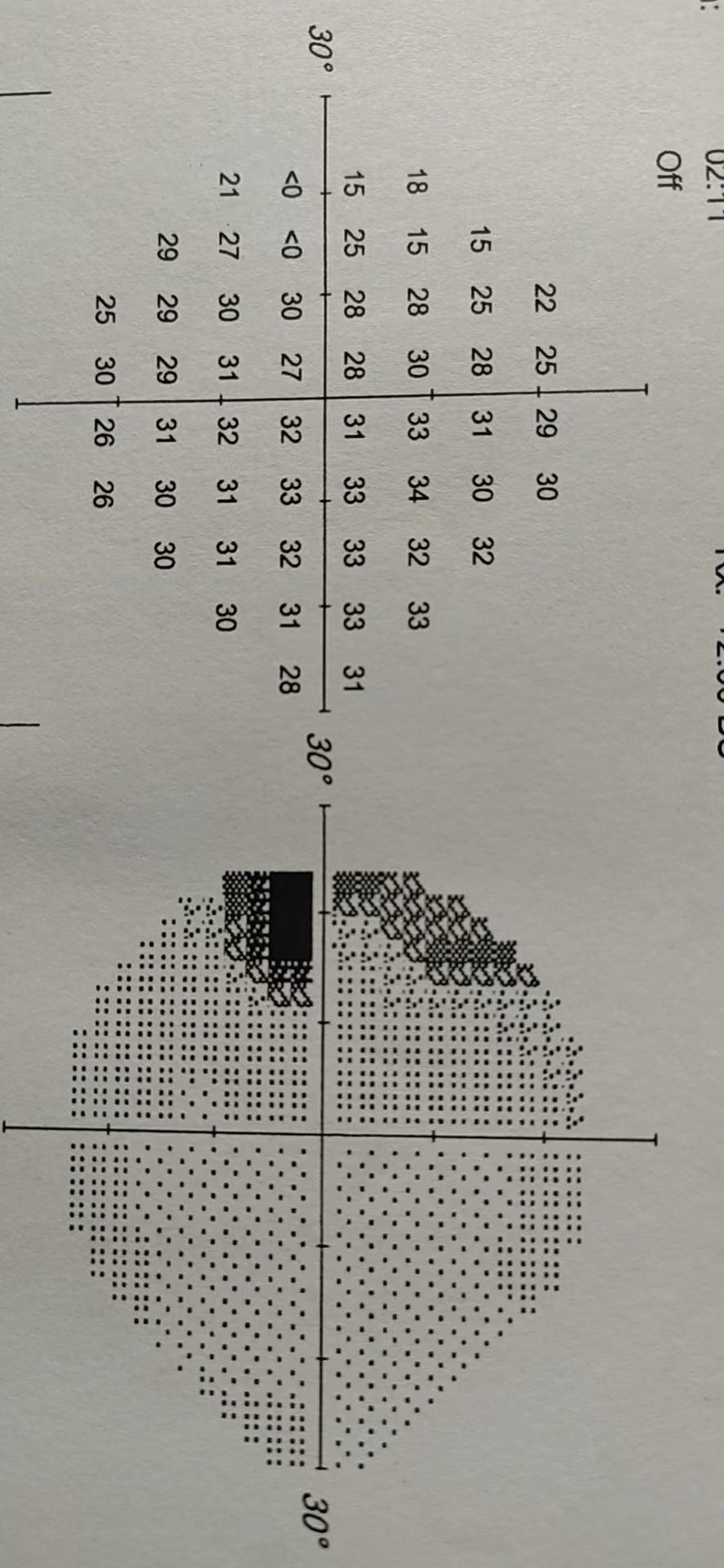

Supplement: Supplementary file 1 — Additional file 1. [file 12902_2023_1345_MOESM1_ESM.zip › leftR3.tif]

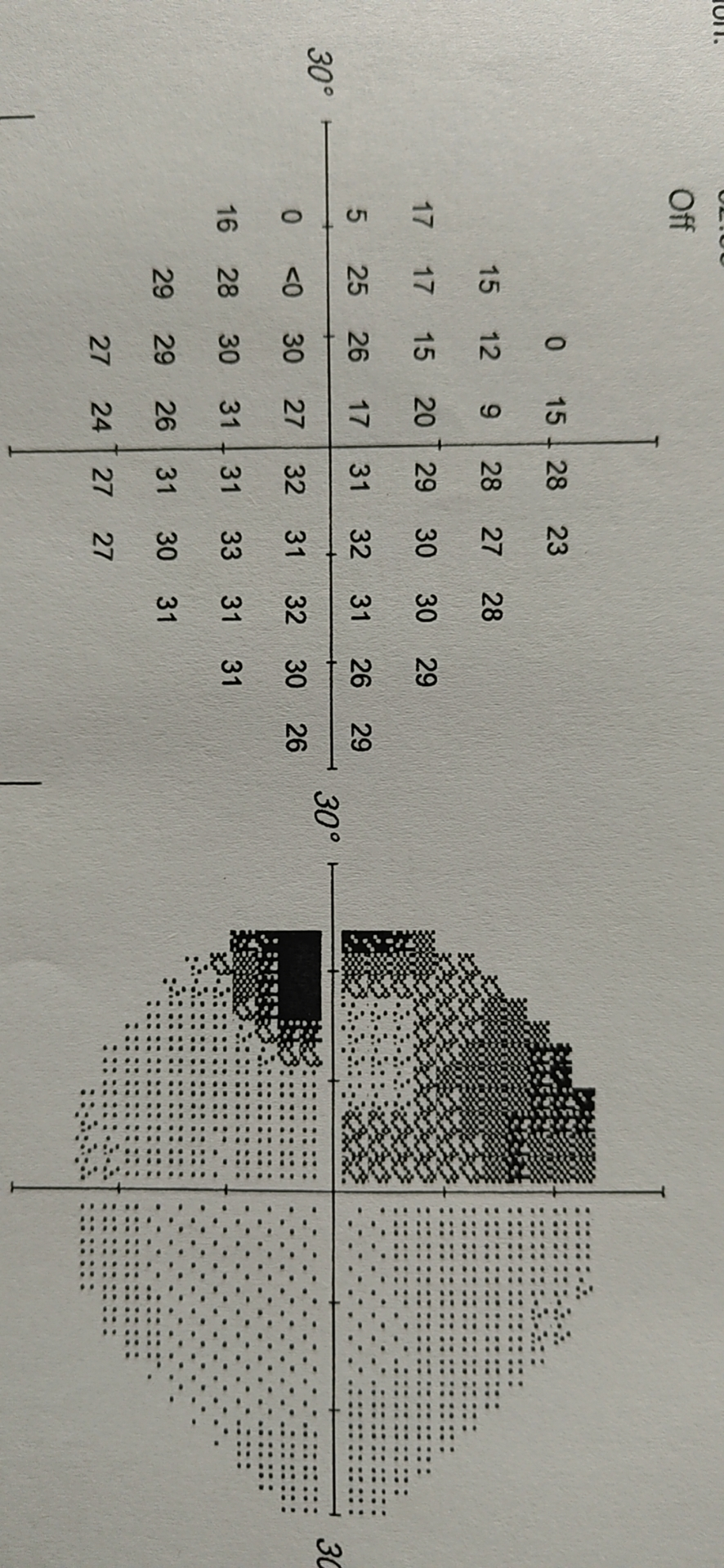

Supplement: Supplementary file 1 — Additional file 1. [file 12902_2023_1345_MOESM1_ESM.zip › rightR3.tif]
